# The influence of empowering team leadership on employees' innovation passion in high-tech enterprises

**DOI:** 10.3389/fpsyg.2022.928991

**Published:** 2022-10-17

**Authors:** Jing Jing, Shujun Wang, Jiaoping Yang, Tianwei Ding

**Affiliations:** ^1^School of Innovation and Practice, Liaoning Technical University, Fuxin, China; ^2^School of Economics and Management, Qingdao University of Science and Technology, Qingdao, China; ^3^School of Business Administration, Liaoning Technical University, Huludao, China

**Keywords:** innovation passion, empowering team leadership, innovation climate based on advantages, innovation self-efficacy, high-tech enterprises

## Abstract

How to stimulate the innovation passion of employees and then improve the innovation performance of enterprises is an important proposition faced by high-tech enterprises. Whether and how to stimulate the innovation passion of employees is of great research value. Based on the social information processing theory, this study takes innovation self-efficiency and innovation climate based on advantages as the path variable and obtains the following conclusions through cross-level analysis with the data of 93 high-tech enterprises as the sample: The empowering team leadership has cross-level direct positive influence and indirect positive influence on innovation passion, and the innovation self-efficiency and innovation climate based on advantages positively adjust the relationship between innovation self-efficiency and innovation passion. At the same time, an innovation climate based on advantages positively moderates the relationship between innovation self-efficacy and innovation passion. This study has some innovations in exploring the function mechanism of empowering team leadership on employees' innovation passion, and the relevant conclusions can guide the innovation management of high-tech enterprises.

## Introduction

High-tech enterprises take technological leadership as their development strategy. How to improve their innovation ability to obtain and maintain competitive advantages is an important source (Anderson et al., 2014). Employees's innovation ability is the core element in the innovation ability system of high-tech enterprises. In the dimension of employee innovation ability, innovation incentive ability is the basic ability (Liu et al., 2019), it is crucial to motivate employees' innovation desire and passion.

According to the concept of work passion (Chen et al., 2020), the innovation passion of employees is a positive inner experience of positive innovation influenced by the external environment, which depends on personality and individual differences, and externally is influenced by leadership behavior and organizational support (Thibault-Landry et al., 2018). The discussion on the relationship between leadership type and employees' innovation has always been a hot topic in academia (Niu et al., 2018; Özsungur, 2019; Wang et al., 2019). Due to the rapid change and uncertainty of the external environment of enterprises, the increasing knowledge level, and the comprehensive quality of employees, empowering leadership behavior has attracted much attention (Wang and Sun, 2018). The research on the innovation relationship between empowering leaders and employees, mainly involves innovation behavior and innovation performance, and the research on the innovation passion of employees has not been reported.

Related research confirmed that employees could effectively improve their work passion when enterprises encouraged them to participate in decision-making and work autonomy (Gao and Jiang, 2019). At this time, employees will show great passion for attaching importance to their job or even enjoying these tasks (Salas-Vallina et al., 2020). This study believes that the knowledge level and demand level of high-tech enterprise employees are higher. If leaders can fully empower and give knowledge workers greater job autonomy, it will be possible to greatly stimulate their enthusiasm for innovation, thereby improving the innovation performance of enterprises.

If empowering team leadership can indeed enhance the innovation passion of employees in high-tech enterprises, then what is their internal mechanism? By reviewing relevant studies, it can be found that these studies are carried out from the perspectives of individual psychology and organizational context, respectively (Lee et al., 2020). Early studies from a single point of view, while recent research began to pay attention to the integration of the two perspectives. Therefore, this paper will also analyze the influence mechanism of empowering team leadership on the innovation passion of employees from the perspective of integration.

Social information processing theory holds that individuals will determine their attitudes and behaviors based on contextual cues (Salancik and Pfeffer, 1978). Empowering team leadership style will release a positive signal to employees, which has an important impact on employees ' attitudes and behaviors. Studies have found that different leadership styles convey different social information to employees and have different impacts on innovation performance (Dai and Lu, 2021). On the one hand, empowering team leadership to express trust and respect for employees and confidence and good expectations in their ability to innovate (Cheong et al., 2019; Gao and Jiang, 2019), increases the motivation of innovation from the perspective of individual psychology. On the other hand, empowering team leadership will send a signal to employees to encourage independent exploration and encourage the development of personality traits (Smallfield et al., 2020; van Knippenberg et al., 2021), which in turn allows employees to perceive a climate of innovation that encourages everyone to take advantage (Yang et al., 2021). As social information processing theory can explain the influence of empowering team leadership on employees' individual psychology and organizational context, it is the theoretical basis of this study.

Compared with the existing research, this study has the following differences: (1) Select employee innovation passion as the target variable, and which can thoroughly interpret how the empowering team leadership promotes employees' innovation from the micro perspective; (2) Analyze the influence mechanism of the organizational situation and individual psychology; (3) Analyze the relative variables based on the team level and individual level and use the cross-level analysis method to explore the influence path of empowering team leadership on employees' innovation passion. In the process of research, innovation self-efficacy was selected as the variable of individual level and individual psychology, and the innovation climate based on advantages was selected as the variable of the organizational situation and team level for mechanism and path analysis.

The rest of this article is as follows. Section 2 introduces the theoretical basis of this paper and constructs the research model of the paper on this basis. Section 3 puts forward the research hypothesis of this paper. Section 4 explains the research methods and data testing process. Section 5 presents the results of our analysis and insightful comments. Section 6 reports the main conclusions and describes the theoretical contributions and practical implications of this paper.

## Research model

### Passion for innovation

Work passion is an emerging topic in the field of organizational and management research because it can well-explain how other variables cannot be thoroughly interpreted in previous studies (Thibault-Landry et al., 2018). In the past decade, it has attracted scholars' much attention (Weng et al., 2020; Smith et al., 2022). At present, by embedding work passion into different situations, some specific concepts have appeared, such as entrepreneur passion (Lex et al., 2022), innovation passion (Kiani et al., 2020), and entrepreneurial passion (Cardon et al., 2017) etc.

Most of the studies related to work passion used it as leading and intermediary variables to explore the effect of work passion, while studies on the pre-factors of work passion are still fewer. In the current research on the causes of work passion, at the individual level, scholars believe that work passion is influenced by self-esteem, autonomy, self-identity, controllability perception, and goal pursuit (Collewaert et al., 2016; Ho et al., 2018). At the team level and organizational level, leadership behavior and leadership style (Afsar et al., 2016), the similarity and differences in member passion (Cardon et al., 2017), and the organizational environment (Ho and Astakhova, 2020) are important variables affecting the teamwork passion and organizational work passion. This study takes the innovation work of high-tech enterprises as the situation, defines innovation passion as the psychological emotion of employees actively producing innovative ideas, and actively seeking new methods, new technologies, and new processes to implement innovative ideas.

### Innovative self-efficacy

Innovation self-efficacy is an individual's belief that they can achieve innovative achievements and master innovative methods (Farmer, 2002). Focusing on innovation self-efficacy, scholars mainly carry out research on the influencing factors, influencing effects, and mediating or moderating effects of innovation self-efficacy. The influencing factors of innovation self-efficacy mainly include organizational factors, leadership factors, work factors and staff factors, such as Wang et al. (2018) studied the innovation climate of innovation self-efficacy, Wang et al. (2014) found that transformational leaders in hotel enterprise change leadership have a positive impact on employees' innovative self-efficacy. The effect of innovation self-efficacy includes employees' innovation behavior, creativity, and innovation performance, such as Michael et al. (2011) in Taiwan enterprise female employees, for example, found that innovation self-efficacy has a positive influence on employees' innovation behavior. Teng et al. (2020) analysis of paired data from supervisors and subordinates found that creative self-efficacy has a greater impact on employees ' creative behavior in an environment of high knowledge sharing. Teng et al. (2020) analyzed the paired data of superiors and subordinates and found that innovative self-efficacy has a greater impact on employees' innovative behavior in an environment of high knowledge sharing.

### Innovation climate based on advantages

Existing researchers have found that the innovative climate has an important impact on employee behavior and employee performance (Thibault-Landry et al., 2018), But, the innovative climate is defined based on the perspective of the employer and organizational needs. Research on organizational climate based on employee needs is relatively lacking (Chen and Huang, 2007), so Van Woerkom and Meyers (2015) proposed a strengths-based psychological climate concept to measure employees' perception of the identification, development, and support of organizations and believes that the improvement of the psychological climate level of the employees based on advantages is equivalent to increasing employees' perception on the organizational recognition, concerns, and use of their own strengths.

This study draws on the concept of psychological climate based on the advantage of Van Woerkom and Meyers (2015), combined with the situation of innovation management, and puts forward the concept of advantage based on innovation climate. It is used to measure the overall climate perception created by the team that can stimulate their innovation advantages. Compared with the psychological climate based on advantages, the innovation climate based on advantages measures the innovation climate at the team level, rather than the psychological climate at the individual level.

### Model based on social information processing theory

According to the social information processing theory, employees will form their own feelings, attitudes, and behaviors based on the information obtained from the organization (team). Using the integration of leadership behavior and employee performance perspective (Cheong et al., 2019), employees perceive empowering team leadership, this information will be based not only on their own individual perspective but also based on the indirect organization perspective. This paper starts from the individual psychology and organizational situation to explore the influence mechanism of empowering leadership on employee innovation passion, which chooses innovation self-efficacy as the individual level of employee psychological experience variable, choose the innovation climate based on advantages as the organization level (team). It can be inferred from the existing research that they have strong explanatory properties in predicting employees' innovation willingness and innovation behavior, so this paper explores whether they have an important impact on the formation of innovation passion.

**Figure 1:**
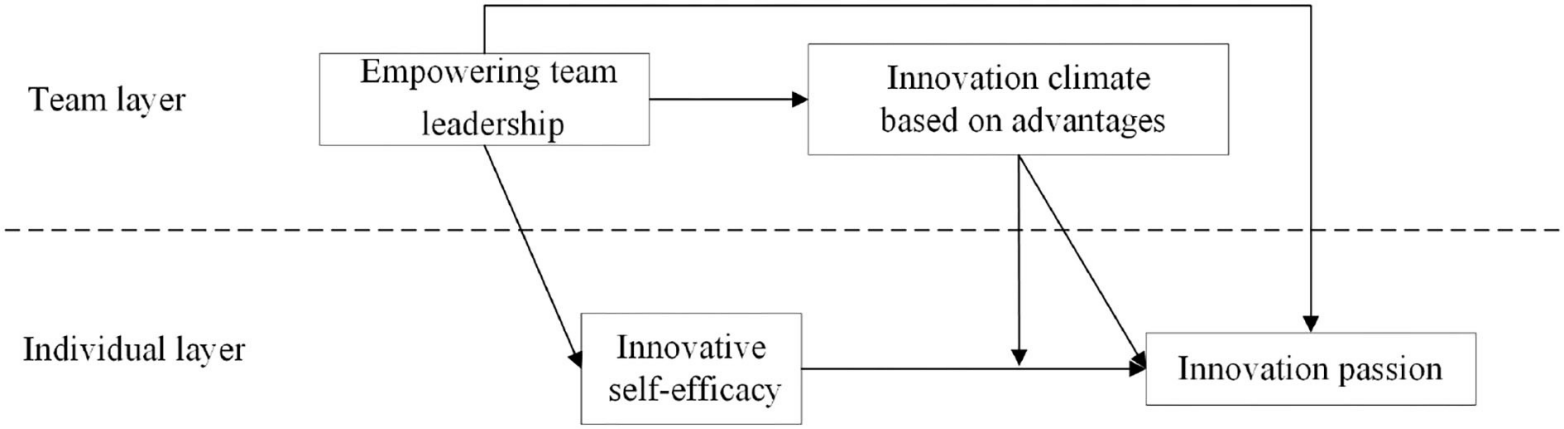
The theoretical model of studies.

According to the meta-analysis of Cui et al. (2019), most existing researchers think that climate variables have affected the production of self-efficacy or play, together with organization climate as an environmental variable, they often have a boundary influence on organization behavior (Schneider and Reichers, 1983), so this paper thinks that advantage-based innovation climate has a regulatory effect on the path of “innovation self-efficacy,” and put forward the theoretical model shown in Figure 1.

Although Van Woerkom and Meyers (2015) psychological climate based on the advantages emphasized more employee demand, for the implementation of incentive staff, in the specific study of Figure 1 whether the innovation climate based on the advantage model can be replaced with an innovation climate, or what is the difference between them is still worth discussion. The empirical research will make a comparative analysis between them to answer this question.

## Research hypotheses

### Empowering team leadership and employees' innovation passion

As mentioned above, innovation passion is working passion embedded in innovation activities. For high-tech enterprises, especially the innovation team, employees' innovation passion is employees strong willingness working state to carry out innovation activities (Thibault-Landry et al., 2018). It is the employees' active production of innovative ideas in the process of work, and actively try to seek new methods, new technology, and new processes to implement the psychological mood of innovative ideas.

The definition of empowering team leadership (Wang et al., 2018) can be seen that it includes the whole process of licensing employees through a series of activities, involving enhancing the work significance of employees, expressing their appreciation for their good performance, and helping employees to eliminate obstacles to improve their performance. For high-tech enterprises, innovative activities are often exploratory, prospective, and risky, therefore, the influence of empowering team leadership on innovation passion can be analyzed from the following aspects: First, empowering team leadership drives employees to continuously improve their self-value and increase the work significance of the employees. To maintain a positive self-evaluation, employees will strive to carry out innovative work. Next, empowering team leadership to convey the recognition and appreciation of their employees greatly improve the work confidence and participation of employees, and promote employees to make forward-looking innovation with greater passion (Farmer, 2002). Once more, empowering team leadership can help employees remove the boundaries between work content and roles and make the innovative thinking of employees not be imprisoned, which creates favorable conditions for employees' innovative behaviors (Xie et al., 2018). Last, empowering team leadership contributes to interaction and sharing of information among team members and helps to reduce the possible risks in the innovation process, which further increases the desire of employees to participate in innovation (Lee et al., 2020). Based on the above analysis, the following assumptions are proposed:

Hypothesis 1. High-tech enterprise empowering team leadership has a positive impact on employees' passion for innovation.

### Mediating role of innovation and self-efficacy

The willingness, ability, and confidence of each employee to innovate is critical for innovation teams in high-tech companies. Therefore, how to improve employees' innovative self-efficacy is highly valued (Park et al., 2021). Many studies have shown that empowering team leadership can indeed improve the perception level of self-efficacy of employees (Cheong et al., 2019). According to the social information processing theory, the influence of empowering team leadership of high-tech enterprises on innovation self-efficacy is mainly reflected in the following aspects: First, empowering team leadership will express their confidence and good expectations about their innovation ability and future innovation performance to their employees, which can make employees full of confidence in their own innovation ability. Next, empowering team leadership will encourage employees to realize the overall value of innovation better in their own work to the organization (team), which makes them actively invest in innovation work. Moreover, when employees discover that empowering team leadership uses their own innovative ideas and methods in decision making, they often perceive their innovative behavior as very valuable. Finally, empowering team leadership put more emphasis on giving employees full power to independent innovation, which is very matched with the characteristics that innovation work needs to break through the routine and dare to take risks.

Existing research points out that innovative self-efficacy has an important impact on employees' innovative behavior (Ho and Astakhova, 2020). According to the above definition of innovation passion, compared with innovation behavior, innovation passion focused more on expressing motivation and willingness. But a consistent conclusion hasn't been achieved on whether there is a correlation between innovation self-efficacy and innovation motivation, or whether innovation self-efficacy affects innovation motivation or innovation motivation affects innovation self-efficacy (Newman et al., 2018). In practice, employees with high competence or strong confidence often show a stronger passion for innovation (Su and Zhang, 2020). Compared with passion, the longer ability is acquired and more stable performance. Ability is more likely to predict passion. Although self-efficacy is a subjective perception of ability, it is based on objective ability. This paper believes that innovative self-efficacy is predictive of innovation passion. The above analysis makes the following assumptions:

Hypothesis 2. Innovation self-efficacy plays a mediating role in the influence of empowering team leadership on employees' passion for innovation.

### Mediating role of innovation climate based on advantages

High-tech enterprises are innovation-oriented compared with other types of enterprises. Its staff management is no longer a rule-based governance model but promotes the establishment of open and inclusive, encouraging autonomy and exploration of the working atmosphere to give full play to the innovative advantages of each employee (Gong et al., 2021). From the perspective of situational empowerment, the management measures of empowering team leadership include moving down, establishing an independent working group, establishing a self-management team, enriching work, etc. Many studies have found that leadership style has an important influence on the climate of organizational innovation (Niu et al., 2018). However, the perspective and degree of influence have their own characteristics. For empowering team leadership, this paper believes that their influence on the innovation climate is mainly based on encouraging independent innovation behavior and encouraging self-advantages, it plays an important role in predicting the innovation climate based on advantages.

From the definition of innovation passion (work passion), it can be found that passion is often manifested as a kind of willingness, emotion, and cognition. According to the perspective of social information processing theory, the information always affects the cognition of innovation work. Therefore, the innovation climate based on advantages can effectively stimulate the innovation passion of employees. In conclusion, the following assumptions are proposed:

Hypothesis 3. Advantage-based innovation climate plays a mediating role in the influence of empowering team leadership on employees' passion for innovation.

### Moderating effect of advantage-based innovation climate

According to the previous analysis, empowering team leadership influences employees' innovation passion through the situational mechanism and psychological mechanism, respectively. Is there any cross influence on these two aspects? This paper believes that the situational variable advantage-based innovation climate may have some influence on the performance of the psychological variable innovation self-efficacy, which can be obtained from the following analysis. Firstly, the innovation climate based on advantage is the perception of the enterprise or team, which can feel the attention and support of the innovation ability, and it is easy to stimulate their positive emotions and improve their ability and confidence. Secondly, the high-tech enterprises or their innovation teams develop various policies and systems for their innovation advantages and encourage employees to have a more positive belief in self-innovation ability.

When high-tech enterprises or their innovation teams create a stimulating climate of individual innovation advantage, some innovation specialties and innovation advantage of the employees are often attached to great importance by the leadership or organization, which is considered that these individual advantages are conducive to enterprise innovation. The stronger the innovation climate based on advantage, the more willingness the employees will be induced to think their own advantages and skills are beneficial to the innovation, which will improve their innovation self-efficiency. On the contrary, when employees think that the enterprises or teams' environment can only act according to the rules and does not encourage the development of personality, they will think that some of their strengths are useless, or cannot contribute to the organizational performance, which will greatly reduce their innovative self-efficiency. The above situation is very common in the actual high-tech enterprise management practice. Many high-tech enterprises are advocating platform-based operations and empowering employees to create a working atmosphere that advocates autonomy and gives full play to individual or team advantages (Tian et al., 2021). Based on the above analysis, the following assumptions:

Hypothesis 4. Advantage-based innovation climate has a positive moderating effect on the mediating effect of innovation self-efficacy.

## Materials and methods

### Study samples and procedures

The survey selected 150 high-tech enterprises from several provinces, mainly distributed in East and North China. The questionnaire distribution and recycling were conducted by site and post. To avoid homologous errors, this study uses leader-member pairing to collect data in a team with the cooperation of human resources departments. Leaders of each team (department) select 5 to 10 subordinates to evaluate their passion for innovation and self-assessment of empowering team leadership. Then the subordinates of each team evaluate the three variables of empowering team leadership, innovative self-efficacy, and innovation climate based on advantages and match the data of leaders and subordinates by coding. Seven hundred and ninety-four questionnaires from 30 enterprises were collected on the spot, and 801 questionnaires from 67 enterprises were collected by mail. Total 97 enterprises, 196 teams, and 1,548 questionnaires.

In the recovered data, the questionnaires with unmatching, improper answers, and obvious response tendencies were excluded. Finally, 162 teams and 1,226 sets of matching questionnaires were obtained from 93 enterprises, with an effective rate of 79.20%. In terms of sample structure, 807 employees are male, accounting for 65.82 %, and 419 are female, accounting for 34.18 %. Most employees are between 20 and 40 years old, accounting for 88.99 %. Approximately 319 people have worked for <5 years, accounting for 26.02 %; 744 people have worked for 5–15 years, accounting for 60.69%; 163 people have worked for more than 15 years, accounting for 13.29%. Among the leaders, 114 were male, accounting for 70.37%, and 48 were female, accounting for 29.63%. All of them had undergraduate education or above, and 35.19% of them had postgraduate education.

### Variable measurement

Most of the measurement items in the scale are selected from mature scales abroad. When making the scale, the three experts first translated it into Chinese and then summarized the feedback, and then translated it back into English to confirm the accuracy of the scale.

Empowering leadership (EL): using Ahearne et al. (2005). The developed scale contains 12 items; typical items include “Leaders often ask my advice when making strategic decisions.” The Cronbach's α value of the scale is 0.899, the composite reliability (CR) is 0.898, and the Average Variance Extracted (AVE) is 0.564.

Innovative Self-efficacy (ISE): Using the scale developed by Karwowski et al. (2013). There are 6 items on the scale, including “I think I can effectively solve even complex problems.” The Cronbach's α value of the scale is 0.907, the Composite reliability (CR) is 0.907, and the Average Variance Extracted (AVE) is 0.599.

Innovation climate based on advantages (ICA): We modified the scale developed by Van Woerkom and Meyers (2015) to adapt to new measurement requirements. The scale includes identifying and developing, appreciating, and using three dimensions, there are 12 items in it. The items include “In this organization, my innovation ability will be appreciated.” The Cronbach's α value of the scale is 0.849, the Composite reliability (CR) is 0.850, and the Average Variance Extracted (AVE) is 0.532.

Innovation Passion (IP): Based on the scale developed by Vallerand and Houlfort (2003), imitate Fang et al. (2017). The two dimensions included 12 items, including “Try new methods and find new things at work.” The Cronbach's α value of the scale is 0.95, the Composite reliability (CR) is 0.895, and the Average Variance Extracted (AVE) is 0.548.

In this study, due to the core task of this study, we treat all variables as single-dimension constructs. In addition, this paper takes employee gender (C1), age (C2), education level (C3), and length of service in the enterprise (C4) as the control variables of this study.

### Common method bias

To reduce the impact of common method bias, first, set up some reverse questions in the questionnaire design to determine whether the logic of the respondents is accurate. Second, it emphasizes that the questionnaire is not used for commercial purposes and is completely anonymous. Finally, the two groups of employees and team leaders complete different questionnaires, as far as possible to reduce the single individual answer the common method bias.

Empowering team leadership, innovation self-efficacy, innovation climate, and innovation passion were combined into a single factor for measurement analysis based on recycled data. The results showed that the single factor model fitting degree or matching effect is not ideal. At the same time, the Harman single-factor test analysis method was used to conduct the exploratory factor analysis of each variable measurement item. The results found that the first factor of the unrotated exploration factor analysis was 24.478%, less than half of the total explanatory variables. In conclusion, the problem of common method bias of the data is not prominent.

### Within-group consistency test

The two variables of empowering leadership and innovation climate based on advantages are the team (enterprise) level. But the sample data comes from individuals. Therefore, aggregated individual-level data at the team level requires a consistency test, as shown in Table 1.

**Table 1:** Team variables Rwg mean value, ICC (1), ICC (2).

	**Rwg average value**	**ICC (1)**	**ICC (2)**
EL	0.972	0.454	0.838
ICA	0.969	0.318	0.748

The average Rwg number of both variables is >0.7, ICC (1) value >0.12, and ICC (2) value >0.7, indicating the high intra-group consistency of sample data. The individual level can be aggregated to the team level by averaging.

### Discrimination validity analysis

Through the comparative analysis of cross-layer confirmatory factors and competition model, the discrimination validity of empowering team leadership, innovative self-efficacy, and innovation climate based on advantages and innovation passion are tested. The results are shown in Table 2. In the four-factor model, χ^2^/df = 1.266, RMSEA = 0.023, GFI = 0.913, CFI = 0.973, TLI = 0.972. All indicators were better than the other models, which indicates that the four-factor model fits the actual data best, namely, the four factors involved in the study had good discrimination validity.

**Table 2:** Cross-layer confirmatory factor analysis.

**Model**	**χ^2^/Df**	**RMSEA**	**NFI**	**TLI**	**CFI**
Single factor model	5.675	0.095	0.483	0.505	0.529
The two-factor model	4.486	0.082	0.591	0.630	0.648
The three-factor model	2.710	0.058	0.753	0.818	0.828
The four-factor model	1.266	0.023	0.885	0.972	0.973

The single-factor model does not distinguish any variables; two-factor model: empowering team leadership, innovative self-efficacy + innovation climate based on advantages + innovation passion; three-factor model: empowering team leadership, innovative self-efficacy + innovation climate based on advantages, innovation passion; four-factor model: this research hypothesis model.

### Descriptive statistical analysis

Table 3 shows the mean, standard deviation, Pearson linear correlation coefficient, and significance level of each variable. The correlation coefficient between constructs is below 0.6, indicating that the measurement data is reliable, indicating that the data homology deviation problem is not serious, the square root of AVE is greater than the correlation coefficient of the corresponding variables, and indicating that the differentiation validity is good and can be used as the purpose of this study.

**Table 3:** Means, standard deviation, and correlation coefficients of the variables.

**Variable**	**Mean**	**Standard Deviation**		**EL**	**ICA**	**ISE**	**IP**
EL	3.574	0.656		**0.751**			
ICA	3.502	0.466		0.472^**^	**0.729**		
ISE	3.528	0.722		0.471^**^	0.285^**^	**0.774**	
IP	3.481	0.483		0.275^**^	0.246^**^	0.328^**^	**0.740**

* Representation p < 0.1,

** representation p < 0.05,

*** It means p < 0.01; the diagonal is AVE square root. The bold values indicate the square root of the Ave.

## Result

The variables in the theoretical model (Figure 1) involve both team and individual levels, and the variables are latent variables. This study uses Mplus7.4 statistical analysis software to construct and analyze hierarchical linear modeling (HLM) to test the previous hypothesis.

### Total effect test

The structural equation model is used to analyze the influence of empowering team leadership on an advantage-based innovation climate and the influence of innovation self-efficacy. The correlation coefficient ICC (1) is 0.111 and 0.06, respectively, so the multi-level regression model for the hypothesis test. The results are shown in Table 4.

**Table 4:** Total effect hypothesis test.

**Administrative levels**			**Parameters estimates**			
**The same level**	**Model: relationship path**		**Estimate**	**S.E**.	**C.R (t)**	***p***
	M1:EL → ICA		0.295	0.033	8.854	0.000
	m1:EL → ISE		0.339	0.040	8.472	0.000
	M2: ISE → IP		0.248	0.035	7.122	0.000
	**Dependent variable**	**Cross-level model**	**γ_00_**	**γ_01_**	**σ^2^**	**τ_00_**
Cross-level		Zero model	3.487***		0.207	0.026
	Innovation passion	M3:EL → IP	2.713***	0.218***	0.208	0.007
		M4:ICA → IP	2.530***	0.296***	0.207	0.013
		m4:ISE → IP	2.879***	0.175	0.208	0.020
		Zero model	3.485***		0.386	0.133
	Innovative self-efficacy	M5:EL → ISE	1.488***	0.564***	0.392	0.004

The ***symbol indicates the value of *p* < 0.01.

In Table 4, to compare the difference between innovation climate (IC) and advantage-based innovation climate, the advantage-based innovation climate is replaced with innovation climate to reanalyze the models m1 and m4.

M3 shows that empowering leadership has a cross-level positive impact on innovation passion, and the impact is significant. Hypothesis 1 is verified. M4 shows that an innovation climate based on advantages also has a cross-level positive impact on innovation passion. When the innovation climate based on advantages is replaced with the innovation climate, m4 shows that the innovation climate does not have a cross-level positive impact on the formation of innovation passion.

### Direct and mediating effect test

Without considering the moderating effect, a cross-level full model of the relationship between the corresponding variables in Figure 1 was established, and the random effect model and the fixed effect model were estimated, respectively. By comparing the random effect model, the direct influence of each variable on the innovation passion was obtained as shown in Table 5.

**Table 5:** Cross-hierarchical models contain all variables.

**Cross-level model (M6)**	**Research model**	**Replace the model**
	**estimate**	**S.E**.	**C.R(t)**	***p***	**Estimate**	**S.E**.	**C.R(t)**	***p***
EL → IP	0.101	0.038	2.642	0.008	0.161	0.039	4.185	0.000
ICA → IP	0.188	0.060	3.150	0.002				
IC → IP					0.021	0.041	0.518	0.604
ISE → IP	0.093	0.036	2.630	0.009	0.114	0.034	3.385	0.001
EL → ICA	0.358	0.054	6.689	0.000				
EL → IC					0.351	0.040	8.862	0.000
EL → ISE	0.563	0.043	13.005	0.000	0.564	0.043	13.270	0.000
EL → ICA → IP	0.067	0.024	2.825	0.005	0.008	0.014	0.530	0.596
EL → ISE → IP	0.053	0.020	2.595	0.009	0.064	0.020	3.296	0.000

As can be seen from the cross-hierarchy model hypothesis testing in Table 5, in addition to having a cross-level direct impact on the passion for innovation (γ = 0.101, *p* < 0.01), it also has an indirect impact on innovation passion through cross-level innovation self-efficacy (γ = 0.053, *p* < 0.01), which has a cross-level indirect influence on the innovation passion through the innovation climate based on advantages at the same level (γ = 0.067, *p* < 0.01). The sum of direct and mediating effects 0.101+(0.067+0.053) = 0.221 is slightly different from the total effect of 0.218 in the M3 model in Table 5, it is due to the influence of the remaining variables of the model.

Interval estimation of the mediation effect of innovation self-efficacy found a 95% confidence interval of [0.013, 0.092], which did not contain zero, so we can determine that innovation self-efficacy act as a mediation variable for the influence of empowering team leadership on innovation passion. Hypothesis 2 was verified. When estimating the intermediary effect of innovation climate based on advantage, we found that the 95% confidence interval was [0.021, 0.114], which does not contain zero. Therefore, the innovation climate based on advantages acted as the moderating variable of the influence of empowering team leadership on innovation passion. Hypothesis 3 was verified.

If the innovation climate based on advantages (ICA) is replaced with the innovation climate (IC), it can be seen from the replacement model that the innovation climate does not appear on the intermediary path of empowering team leadership and innovation passion.

### Test of moderating effect

**Figure 2:**
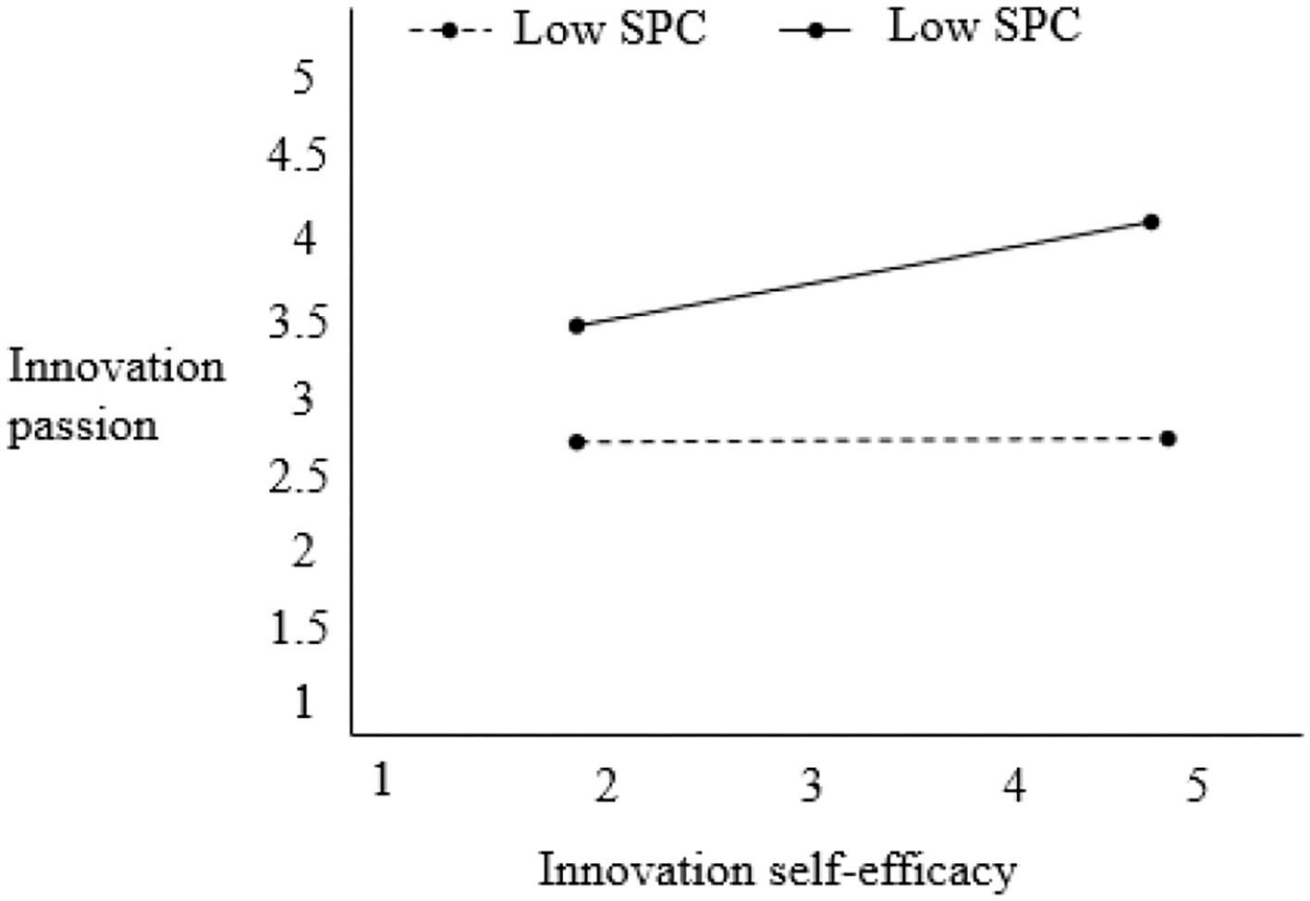
Regulating effect of the innovation climate based on advantages.

When the psychological climate based on innovative psychological advantage was added as a regulatory variable to the model, the individual level variable relationship (β: ISE → IP) was regulated by AIC (γ = 0.114, *p* < 0.01), and the 95% confidence interval [0.054, 0.173], which does not contain 0, so that the adjustment effect was valid, assuming hypothesis 4 was verified. As can be seen from Figure 2, when the innovation climate based on advantages is at a high level, innovation self-efficacy and innovation passion show a significant positive correlation (β = 0.158, *p* < 0.01). When the advantage-based innovation climate is at a low level, the positive correlation between innovation self-efficacy and innovation passion is no longer significant (β = 0.036 n.s.).

The bootstrap method set resampling to 2,000 times to obtain the following analysis results: when the innovation climate based on advantages was low, the 95% confidence interval was [-−0.047, 0.032], and 95% [0.0073, 0.153].

When the innovation climate based on advantages (ICA) is replaced with innovation climate (IC), the model shows that the individual-level variable relationship (β: ISE → IP) is regulated by IC is (γ = 0.254, *p* < 0.01), 95% confidence interval is [0.143, 0.364], which does not contain 0, it can determine the adjustment effect is true.

Overall analysis, the hypothesis of this study is supported, namely the influence of empowering team leadership on innovation passion mechanism has the following path: empowering team leadership → innovation passion, empowering team leadership → innovation self-efficacy → innovation passion, empowering team leadership → innovation climate based on advantages → innovation passion, empowering team leadership → innovation climate based on advantages × innovation self-efficacy → innovation passion. When the innovation climate based on advantages is replaced with the innovation climate, the innovation climate no longer appears in the intermediary path, but the innovation climate moderates the intermediary effect of innovation self-efficacy.

## Discussion

This study takes the data of 162 teams from 93 high-tech enterprises in China as samples, and uses the cross-level structural equation model analysis method to find that: Empowering team leadership has a positive impact on innovation passion, in which innovative self-efficacy and innovation climate based on advantages play a partial mediating role. The innovation climate based on advantages positively regulates the relationship between innovation self-efficacy and innovation passion. The specific conclusions are as follows:

(1) Empowering team leadership in the high-tech enterprise have a significant positive impact on employees' innovation passion, including both direct and indirect effects. According to the theoretical analysis, the empowering team leadership mode increases the autonomy and participation of employees through empowerment and realizes the unity of responsibilities and rights and tasks and interests to a certain extent, then effectively stimulating employees' passion for innovation (Xie et al., 2018; Wang et al., 2019). The research in this paper shows that empowering team leadership in high-tech enterprise create an innovation climate based on advantages and stimulate innovation self-efficacy, thus improving employees' innovation passion.
(2) Innovation self-efficacy and innovation climate based on advantages can partly explain the influence of empowering team leadership on employees' innovation passion. The theoretical analysis and empirical test of this paper show that, on the one hand, empowering team leadership increases innovative self-efficacy by giving employees confidence and expectations for innovation (Teng et al., 2020; Park et al., 2021). On the other hand, the innovation climate based on advantages is improved by encouraging self-advantage, and the innovation self-efficacy and the innovation atmosphere based on advantages can effectively stimulate the innovation passion as intermediary variables.
(3) The innovation climate and innovation climate based on advantages can explain the influence of empowering leadership on employees' innovation passion, but the action mechanism is not the same. Empirical research in this study found that although empowering team leadership has a positive predictive effect on both innovation climate based on advantages and innovation climate, their influencing mechanisms on innovation passion are different. Innovation climate based on advantages has a direct influence on innovation passion and moderates the relationship between innovation self-efficacy and innovation passion, while innovation climate only moderates the relationship between innovation self-efficacy and innovation passion as environment and boundary (Yang et al., 2021).

### Theoretical contribution

Compared with the current research, this study has made the following theoretical progress, which has important theoretical significance for enriching similar research.

First, the formation mechanism of employees' innovative passion is analyzed with empowering team leadership as the influencing factor. In recent years, work passion and innovation passion have been used as atomic constructs in related fields to analyze many organizational behavior problems and phenomena (Gielnik et al., 2015; Cardon et al., 2017). However, most studies use it as a pre-factor or path variable (Türk et al., 2019; Yukhymenko-Lescroart and Sharma, 2019), and there are few studies on its formation mechanism (Breu and Yasseri, 2022). This paper analyzes the formation mechanism of innovation passion with empowering team leadership as the pre-factor.

Second, from the two paths of individual psychology and organizational context, the cross-level analysis method is used to explore the influence mechanism of empowering team leadership on employees ' innovative passion based on the integrated perspective. Most of the studies on the relationship between leadership style and employee innovation were carried out from the perspectives of individual psychology and organizational context (Wang et al., 2021; Xu et al., 2022). This study integrates two perspectives, each choosing a path variable to analyze the impact of empowering team leadership on employees' innovative passion (Individual psychological perspective is innovative self-efficacy; Innovation climate based on advantages from the perspective of organizational context). Since the relevant constructs in the study involve the organizational level and the individual level, the cross-level analysis method (Wood et al., 2021) is selected and the research shows that the design is rational.

There are still many limitations and shortcomings in this study. First, this study uses cross-sectional data. Although there are many theories to support the causal relationship between variables, it is still very limited to infer a causal relationship from data analysis. Future research can use quasi-experimental or experimental research methods to further do robustness test. Secondly, this study only controls the individual level variables such as employee gender, age, education, and length of service in the enterprise, but not the team level variables. The path of innovation climate based on advantages may not be robust enough, and future research should be supplemented.

### Practical implications

First, for high-tech enterprise employees, especially the innovation team staff, it is recommended to fully empower them. Empowering team leadership is an effective way to stimulate employees' innovative passion. On the one hand, empowering team leadership can increase employees' innovative self-efficacy. On the other hand, empowering team leadership can create an innovative atmosphere based on advantages for the organization. There are a lot of enterprise cases that can be proved. Such as Haier through the implementation of the platform + small micro organizational model to achieve empowering leadership change, giving each innovation and entrepreneurship small micro enough autonomy, greatly stimulating the work passion of employees and teams.

Secondly, for high-tech enterprises, how to increase employees' willingness to innovate, ability and self-confidence are very important. Enterprises should give employees the opportunity to exercise and improve themselves, thereby increasing their innovative self-efficacy. In addition, companies should be good at discovering each employee's expertise and interests and configure them for the most appropriate positions and project teams. Enterprises should regularly or irregularly carry out employee innovation ability training, broaden their innovative horizons, stimulate their innovative thinking and make employees believe that they can innovate.

Finally, to improve the innovation ability of high-tech enterprises and stimulate employees' innovation passion, enterprises should create a climate to encourage innovation and give employees certain independent decision-making power. On the one hand, creating a climate that encourages individual advantages and gives employees more job autonomy will help enhance their sense of ownership and belonging to the company, thereby stimulating the endogenous motivation of employees to innovate. On the other hand, creating an innovative atmosphere based on advantages will help encourage employees to actively express their ideas and fully demonstrate their professional expertise, to produce solutions that create value.

## Data availability statement

The raw data supporting the conclusions of this article will be made available by the authors, without undue reservation.

## Ethics statement

The protocol was approved by an Institutional Review Board of Liaoning Technical University of China. All subjects read informed consent before participating this study and voluntarily made their decision to complete surveys.

## Author contributions

With the cooperation of SW, JY, and TD this paper has got the convincing survey data and reach the current solution. All authors contributed to the article and approved the submitted version.

## Funding

This work was supported by scientific research fund project of Education Department, Liaoning Provincial (LJ2020QNW003).

## Conflict of interest

The authors declare that the research was conducted in the absence of any commercial or financial relationships that could be construed as a potential conflict of interest.

## Publisher's note

All claims expressed in this article are solely those of the authors and do not necessarily represent those of their affiliated organizations, or those of the publisher, the editors and the reviewers. Any product that may be evaluated in this article, or claim that may be made by its manufacturer, is not guaranteed or endorsed by the publisher.
